# Carcinoembryonic antigen trajectory predicts pathological complete response in advanced gastric cancer after neoadjuvant chemotherapy

**DOI:** 10.3389/fonc.2025.1525324

**Published:** 2025-02-10

**Authors:** Yonghe Chen, Dan Liu, Kaikai Wei, Yi Lin, Zhong Wang, Qian Sun, Huashe Wang, Junsheng Peng, Lei Lian

**Affiliations:** ^1^ Department of General Surgery, The Sixth Affiliated Hospital, Sun Yat-sen University, Guangzhou, China; ^2^ Guangdong Provincial Key Laboratory of Colorectal and Pelvic Floor Diseases, The Sixth Affiliated Hospital, Sun Yat-sen University, Guangzhou, China; ^3^ Biomedical Innovation Center, The Sixth Affiliated Hospital, Sun Yat-sen University, Guangzhou, China; ^4^ Department of Laboratory Science, The Second Affiliated Hospital of Guangzhou University of Chinese Medicine, Guangzhou, China; ^5^ Department of Radiology, The Sixth Affiliated Hospital, Sun Yat-sen University, Guangzhou, China; ^6^ School of Nursing, Sun Yat-sen University, Guangzhou, China

**Keywords:** gastric cancer, carcinoembryonic antigen, trajectory analysis (TA), pathological complete response, neoadjuvant chemotherapy

## Abstract

**Aims:**

This study aims to develop a simple, clinically applicable classification system to predict pCR based on carcinoembryonic antigen (CEA) trajectory during NAC.

**Methods:**

This study included 366 AGC patients who received NAC followed by radical gastrectomy. CEA levels were measured before, during, and after NAC, with changes classified into three trajectory types: Type I (>=80% decline), Type II (>=40% but <80% decline), and Type III (<40% decline or increase). We analyzed associations between these CEA trajectories, pCR, lymph node remission, and survival.

**Results:**

pCR was achieved in 10.4% (38/366) of patients. pCR rates were significantly higher in Type I (41%) and Type II (15.8%) trajectories compared to Type III (6.7%). Lymph node remission also correlated with CEA trajectories, with Type I having the highest proportion of ypN0 (79.2%). Multivariate analysis identified CEA trajectory subtypes and tumor differentiation as independent predictors of pCR. This classification system proved robust across subgroups. Although no significant differences in overall survival were observed between subtypes, higher initial CEA levels were associated with worse survival.

**Conclusion:**

The trajectory of CEA change during NAC is a promising predictor of pCR in AGC. This simple and accessible classification system may facilitate personalized surgical strategies for patients with AGC.

## Introduction

Gastric cancer, a leading cause of cancer-related mortality globally, is often diagnosed at advanced stages, particularly in China (1, 2). Surgical resection offers the only potential cure for advanced disease, but high recurrence rates persist due to incomplete tumor removal (3). Neoadjuvant chemotherapy (NAC) has been incorporated into treatment to address this issue by downstaging tumors, facilitating complete resection, and improving survival (4, 5). However, patient responses to NAC vary due to the heterogeneous nature of gastric cancer cells (6, 7). The current gold standard for assessing response to neoadjuvant chemotherapy (NAC) in gastric cancer is through pathological examination of the surgically resected specimen, with the most desirable outcome being a pathological complete response (pCR), defined as the absence of residual tumor cells in both the primary tumor and surrounding lymph nodes (8). However, this assessment can only be made after surgical resection. Preoperative evaluation of NAC response is crucial for individualized treatment plans (9). Patients demonstrating a favorable response may be suitable candidates for function-preserving surgeries like proximal or distal gastrectomy, which preserve gastric function and quality of life (10). On the other hand, those with a poor response may necessitate total gastrectomy to ensure comprehensive lymph node removal and to reduce recurrence risk (11). Therefore, accurate preoperative prediction of NAC response is essential for optimizing surgical strategies. However, accurate assessment of pathological response before surgery is difficult, the most common approach in clinical practice is to compare the computed tomography (CT) scans before and after the NAC to evaluate response according to RECIST guidelines (12), but this method has proven unreliable. Discrepancies between RECIST-determined responses and actual pathological findings are frequently encountered and can be misleading, warranting new method (13).

Carcinoembryonic antigen (CEA), a widely used tumor marker in gastrointestinal malignancies, is associated with tumor staging, burden, and load (14). While monitoring CEA levels during chemotherapy and post-surgical follow-up is recommended (15), the precise relationship between the magnitude of CEA decline and treatment response remains unclear. Quantifying this relationship is crucial for clinical practice as it could inform decision-making regarding surgical approaches and potentially predict pCR.

To address this problem, we conducted a retrospective analysis of CEA levels during neoadjuvant chemotherapy (NAC). Our study aimed to investigate the association between the dynamic changes in CEA values during chemotherapy and the pathological response, aiming to quantify the relationship between CEA decline and pCR.

## Methods

### Study population

Inclusion criteria:

➢ Age 18-80 years
➢ Histologically confirmed gastric or esophago-gastric junction adenocarcinoma
➢ Underwent preoperative neoadjuvant chemotherapy (NAC) followed by radical gastrectomy
➢ At least three CEA examinations during NAC

Exclusion criteria:

➢ Received concurrent radiotherapy or targeted therapy during NAC
➢ Insufficient clinical staging information or uncertain distant metastasis
➢ Secondary concurrent malignancy

These criteria ensured the selection of a well-defined study population and maintained data quality and relevance for analysis.

### CEA data collection and trajectory classification

CEA were measured at three time points in all patients undergoing neoadjuvant chemotherapy (NAC): before NAC, during NAC (at an intermediate timepoint), and after NAC (before the resection surgery). CEA trajectories were constructed by connecting these three values for each case, depicting the dynamic changes in CEA levels under the influence of NAC. CEA trajectories were classified based on a combination of clinical relevance, statistical significance, and practical applicability in the clinical setting.

### Pre-intervention staging

Prior to treatment initiation, the clinical stage of each patient was assessed using enhanced thoracic-abdominal-pelvic computed tomography and/or endoscopic ultrasonography, adhering to the American Joint Committee on Cancer (AJCC) staging criteria (16).

### Chemotherapy regimens

The following chemotherapy regimens were employed in this study:

**FLOT**: Docetaxel, oxaliplatin, and fluorouracil; administered every 2 weeks.

**SOX**: Oxaliplatin, Tegafur Gimeracil Oteracil Potassium Capsule (S-1); administered every 3 weeks.

**XELOX**: Oxaliplatin, Capecitabine; administered every 3 weeks.

**FOLFOX**: Oxaliplatin, and fluorouracil; administered every 2 weeks.

**Others**: In some cases, modified regimens were utilized due to patient allergies or other individual factors. These modifications primarily involved biweekly docetaxel with fluorouracil/S1 or mono-agent oral therapy of capecitabine.

### Pre-operative assessment

After the completion of NAC, the resectability of the primary tumor was re-confirmed by enhanced thoracic-abdominal-pelvic computed tomography.

### Surgical resection

All patients underwent curative gastrectomy (total or subtotal) with standard D2 lymphadenectomy. Prior to resection, a thorough abdominal exploration was performed to determine the status of peritoneal metastasis.

### Pathological assessment

Resected specimens were examined for pathological staging and tumor regression grade (TRG) following neoadjuvant therapy. PCR was defined as the absence of residual tumor cells in both the primary tumor site and dissected lymph nodes.

### Follow-up

Follow-up assessments during the first two years included appointments per 3 months for the first six months, followed by appointments every 6 months. Each visit included a comprehensive review of medical history, physical examination, blood tests, biochemical analyses, and CT scans. If a patient failed to attend a scheduled appointment, the hospital’s follow-up office would collect information on their health and survival status through telephone calls or mail. Overall survival (OS) was defined as the duration from surgery to death or the final follow-up date.

### Data analysis

The Kolmogorov-Smirnov test and normal probability plots were used to assess data normality. Parameters with non-normal distributions were reported as medians (interquartile ranges) and analyzed using non-parametric tests (Mann-Whitney or Kruskal-Wallis, as applicable). For normally distributed data, means ± standard deviations were calculated and analyzed with Student’s t-test. Categorical variables were assessed with the chi-square test. Survival differences were evaluated using the Kaplan-Meier method, and hazard ratios were determined through Cox regression. All analyses were conducted in R v4.3.1 (The R Foundation), with statistical significance set at p < 0.05.

## Results

### Patient characteristics

From Feb 2013 to Nov 2020, we identified 366 eligible patients who received neoadjuvant chemotherapy followed by D2 radical gastrectomy. As shown in **Table 1**, the cohort consisted mostly of male patients (271/366, 74%), with a median age of 65 years. The tumors were predominantly poorly differentiated adenocarcinoma (269/366, 73.5%) in advanced stages, with all cases exceeding clinical stage T3 and more than half being stage N2 or above.

**Table 1:** Patients baseline characteristics of different trajectory subtypes.

Characteristic (%)	Overall (n=366)	Type I Marked Decline (n=24)	Type II Moderate Decline (n=57)	Type III Limited/No Decline (n=285)	p-value
Sex					
*Female*	95 (26.0)	3 (12.5)	10 (17.5)	82 (28.8)	0.063
*Male*	271 (74.0)	21 (87.5)	47 (82.5)	203 (71.2)	
**Age**	65 [56, 70]	67 [64, 72]	64 [58, 68]	65 [55, 70]	0.202
Location					
*Lower*	161 (44.0)	12 (50.0)	17 (29.8)	132 (46.3)	0.088
*Middle*	64 (17.5)	2 (8.3)	10 (17.5)	52 (18.2)	
*Upper*	141 (38.5)	10 (41.7)	30 (52.6)	101 (35.4)	
Differentiation					
*Moderate*	80 (21.9)	6 (25.0)	18 (31.6)	56 (19.6)	0.016
*Poor*	269 (73.5)	15 (62.5)	34 (59.6)	220 (77.2)	
*Well*	17 (4.6)	3 (12.5)	5 (8.8)	9 (3.2)	
Lauren Type					
*Diffuse*	195 (53.3)	10 (41.7)	19 (33.3)	166 (58.2)	0.001
*Intestine*	52 (14.2)	7 (29.2)	15 (26.3)	30 (10.5)	
*Mix*	119 (32.5)	7 (29.2)	23 (40.4)	89 (31.2)	
Clinical T stage					
*T3*	244 (66.7)	18 (75.0)	40 (70.2)	186 (65.3)	0.482
*T4a*	89 (24.3)	4 (16.7)	15 (26.3)	70 (24.6)	
*T4b*	33 (9.0)	2 (8.3)	2 (3.5)	29 (10.2)	
Clinical N stage					
*N0*	12 (3.3)	1 (4.2)	1 (1.8)	10 (3.5)	
*N1*	145 (39.6)	9 (37.5)	17 (29.8)	119 (41.8)	0.203
*N2*	166 (45.4)	8 (33.3)	32 (56.1)	126 (44.2)	
*N3*	43 (11.7)	6 (25.0)	7 (12.3)	30 (10.5)	
Chemo Regimen					
*DOF*	214 (58.5)	20 (83.3)	38 (66.7)	156 (54.7)	0.220
*OF*	23 (6.3)	1 (4.2)	2 (3.5)	20 (7.0)	
*OS*	101 (27.6)	3 (12.5)	13 (22.8)	85 (29.8)	
*Others*	9 (2.5)	0 (0.0)	2 (3.5)	7 (2.5)	
*OX*	19 (5.2)	0 (0.0)	2 (3.5)	17 (6.0)	
**Neoadjuvant cycles**	4 [4, 4]	4 [4, 4]	4 [4, 5]	4 [3, 4]	0.509
Laparoscopic					
*Laparoscopic*	304 (83.1)	20 (83.3)	46 (80.7)	238 (83.5)	0.875
*Open*	62 (16.9)	4 (16.7)	11 (19.3)	47 (16.5)	
Resection Extent					
*Distal*	147 (40.2)	10 (41.7)	13 (22.8)	124 (43.5)	0.045
*Proximal*	3 (0.8)	0 (0.0)	0 (0.0)	3 (1.1)	
*Total*	216 (59.0)	14 (58.3)	44 (77.2)	158 (55.4)	
Tumor clearance					
*R0*	329 (89.9)	22 (91.7)	48 (84.2)	259 (90.9)	0.384
*R1*	34 (9.3)	2 (8.3)	9 (15.8)	23 (8.1)	
*R2*	3 (0.8)	0 (0.0)	0 (0.0)	3 (1.1)	
ypT stages					
*T0*	39 (10.7)	10 (41.7)	9 (15.8)	20 (7.0)	<0.001
*T1*	35 (9.6)	2 (8.3)	5 (8.8)	28 (9.8)	
*T2*	40 (10.9)	3 (12.5)	4 (7.0)	33 (11.6)	
*T3*	220 (60.1)	7 (29.2)	33 (57.9)	180 (63.2)	
*T4a*	27 (7.4)	2 (8.3)	5 (8.8)	20 (7.0)	
ypN stage					
*N0*	173 (47.3)	19 (79.2)	20 (35.1)	134 (47.0)	0.001
*N1*	68 (18.6)	2 (8.3)	15 (26.3)	51 (17.9)	
*N2*	55 (15.0)	1 (4.2)	4 (7.0)	50 (17.5)	
*N3*	70 (19.1)	2 (8.3)	18 (31.6)	50 (17.5)	
**Harvested lymph node**	31 [21, 40]	30 [17, 41.50]	31 [23, 44]	31 [21, 39]	0.605

### Neoadjuvant chemotherapy

More than half of patients (214/366, 58.5%) receive the FLOT regimen as neoadjuvant chemotherapy regimen, and approximately one third (143/366, 39.1%) receive doublet regimen with oxaliplatin plus fluorouracil (or its analogue), such as the FOLFOX, SOX and XELOX regimen. A total of 9 patients received modified regimen with docetaxel and fluorouracil/S1 due to platinum allergies or other individual factors. A median of 4 cycles were deployed. Eventually, 10.4% (38/366) patients achieve pathological complete response.

### CEA trajectory analysis and classification

**Figure 1:**
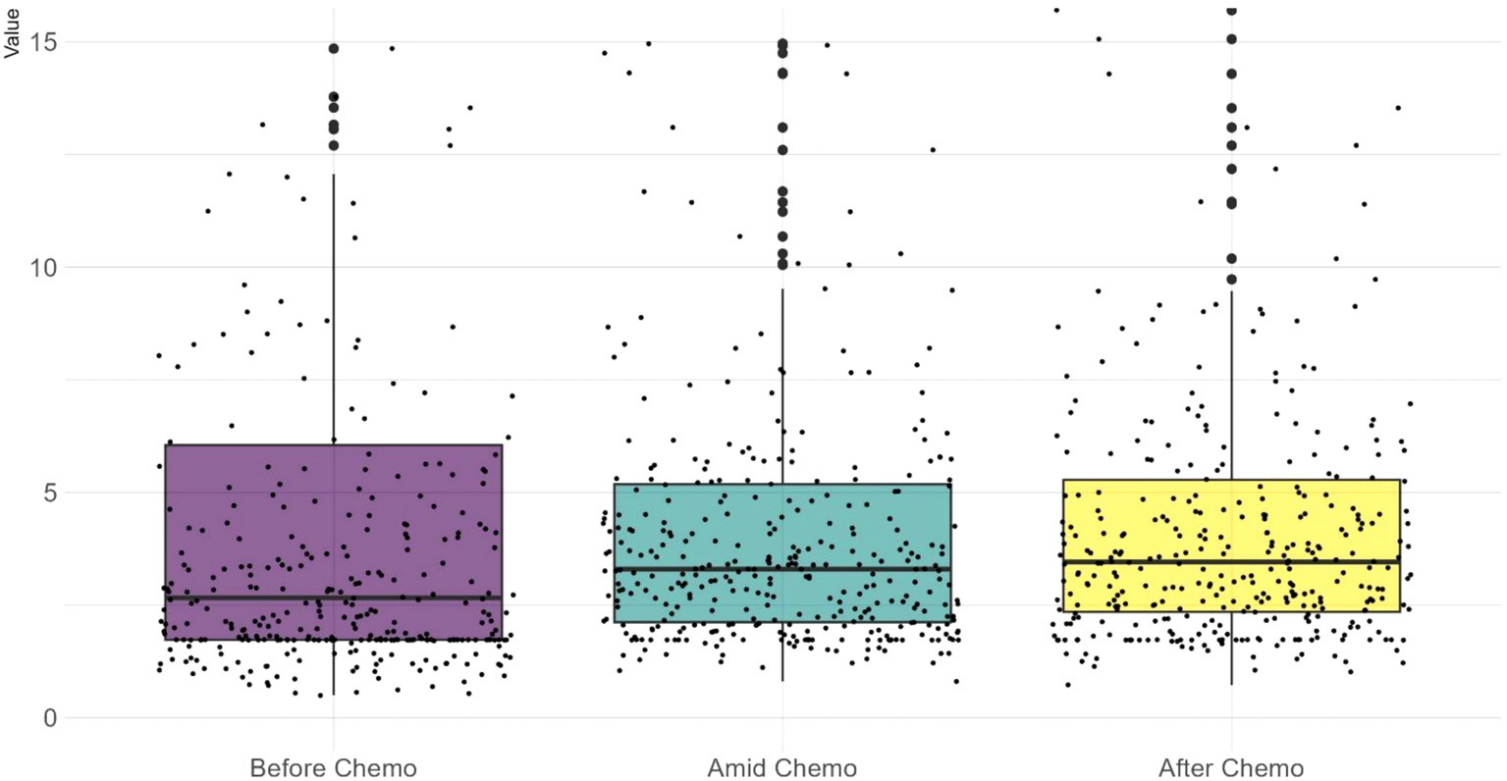
A scattered boxplot showing CEA test values across the chemotherapy timeline. Each dot represents a case. Before chemotherapy, test values exhibit a wider range and a lower median. During chemotherapy, the median slightly increases while the range narrows. Wilcoxon test shows no significant difference between these three datasets.

**Figure 2:**
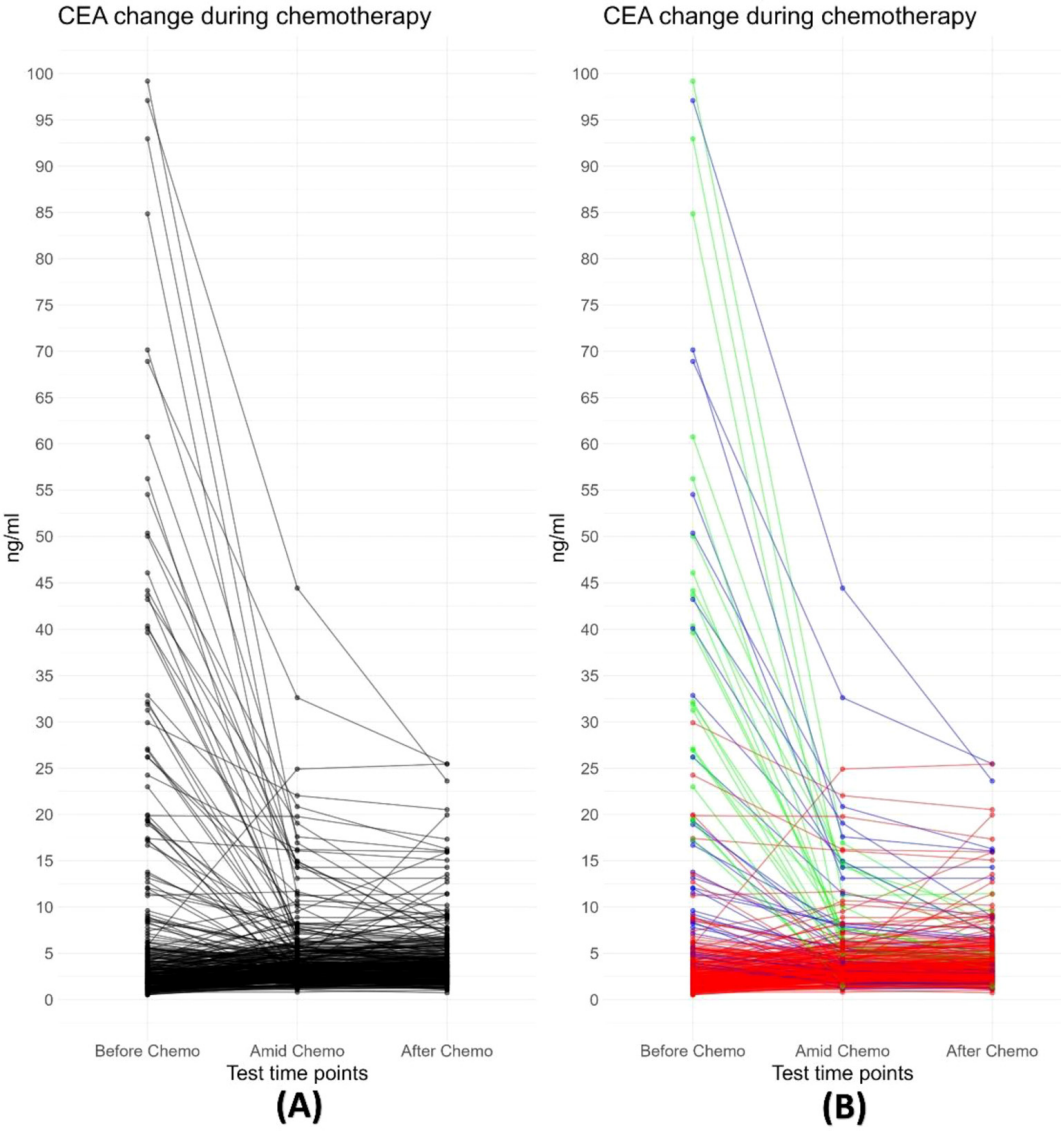
**(A)** Trajectory plot depicting changes in CEA values during neoadjuvant chemotherapy. Each black line represents an individual case. The plot demonstrates a decline in CEA values for most cases during chemotherapy, particularly those with abnormally high pre-treatment values. **(B)** All trajectories are subgrouped into three classes based on the change in CEA values following neoadjuvant chemotherapy: Type I (“Free fall”), defined as ≥80% decrease; Type II (“Slippery slope”), defined as ≥40% but <80% decrease; and Type III (“Plateau or Uphill”), defined as <40% decreases or any increase in CEA values.

**Figure 3:**
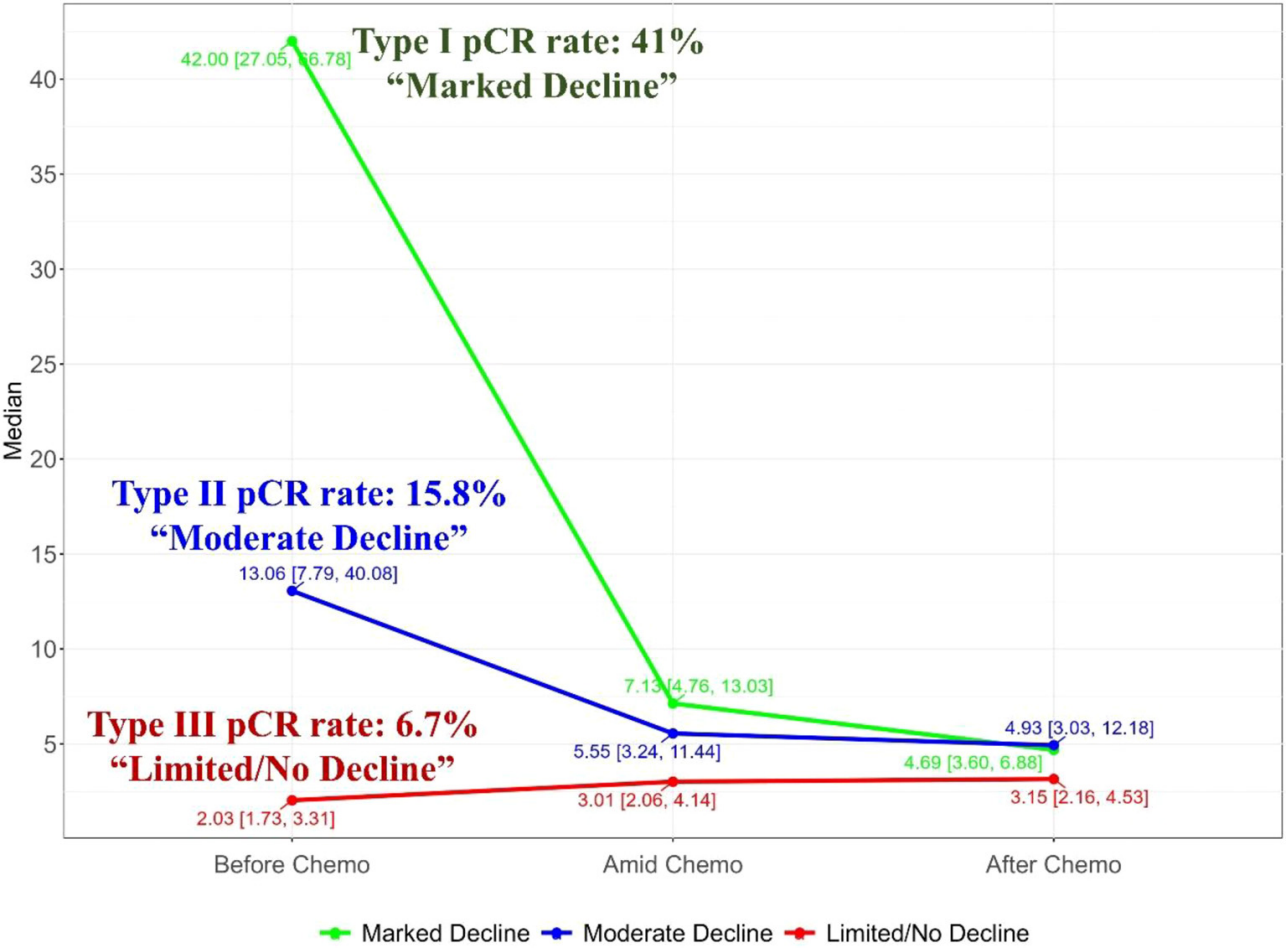
Merged trajectory plot depicting the median CEA value changes of the three trajectory classes, showing significant discrepancy between the trajectory classes.

As shown in **Figure 1**, before chemotherapy, the median CEA value were 2.66 ng/ml, with a wide range from 0.5ng/ml to 1210.4ng/ml. 29.5% (108/366) had an elevated CEA level (5 ng/ml as the threshold for abnormal elevation). **Figure 2** depicts the trajectories of the CEA levels during chemotherapy, each lines represent a case and each dot represent the test value at a specific time point, highlighting the dynamic change of CEA throughout the process of therapy. 26.2% (96/366) patients had a declining CEA value during NAC. According to the dynamic change of CEA value, the trajectories were categorized into 3 types: Type I (“Marked decline”), defined as > =80% decrease; Type II (“Moderate decline”), defined as > =40% but <80% decrease; and Type III (“Limited/No decline”), defined as <40% decreases or any increase in CEA values. As shown in **Figure 3**, the changing patterns of these three types of trajectories is significantly different, with an F value of 20.335 and a p value <0.01 in the ANOVA analysis. This indicates that the CEA values at different time points vary significantly across different trajectory types.

### CEA trajectories and pathological response

**Figure 4:**
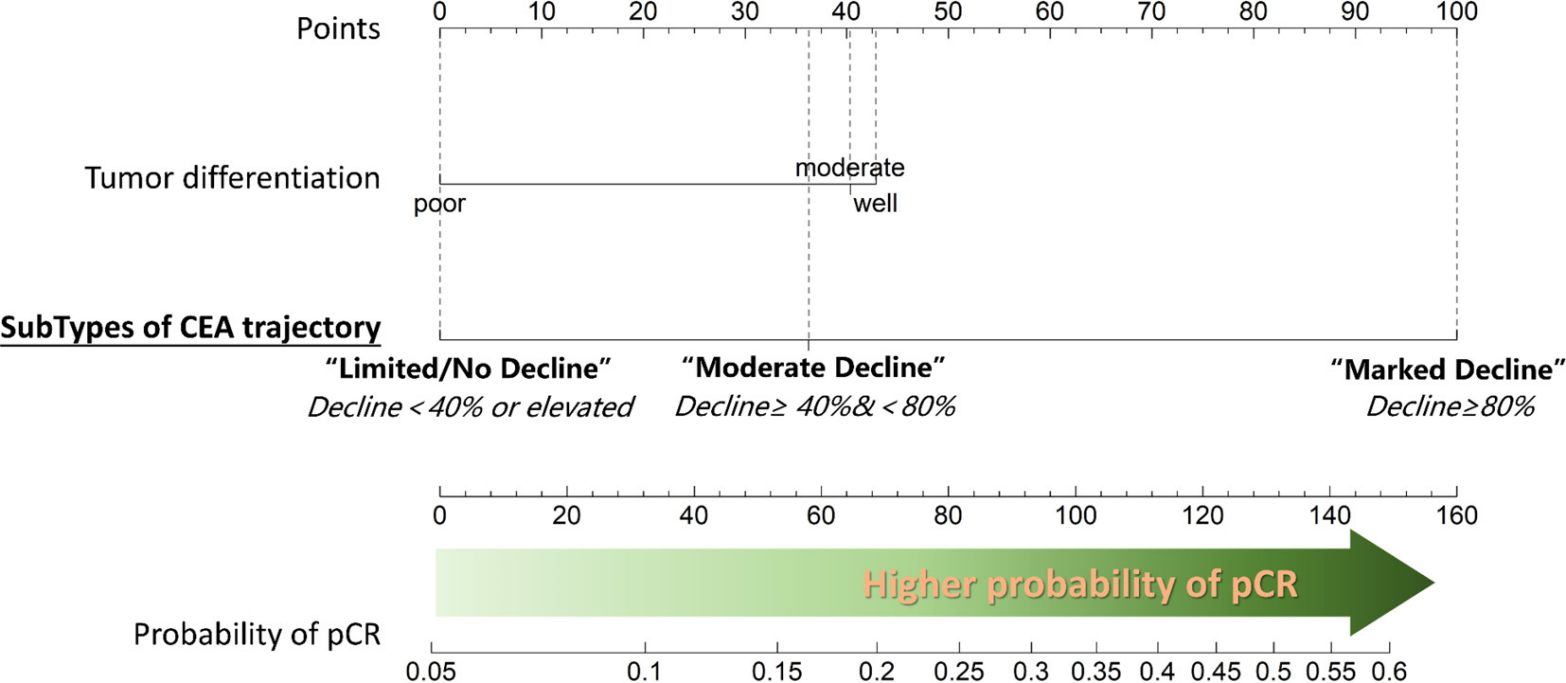
Nomogram depicting the results of multivariable logistic regression analysis for predicting pathological complete response (pCR). Tumor differentiation and the change in carcinoembryonic antigen (CEA) levels during neoadjuvant chemotherapy (NAC) emerged as the only two significant predictors of pCR. Notably, better tumor differentiation and a substantial decrease in CEA values during NAC are associated with higher probability of achieving pCR.

**Figure 5:**
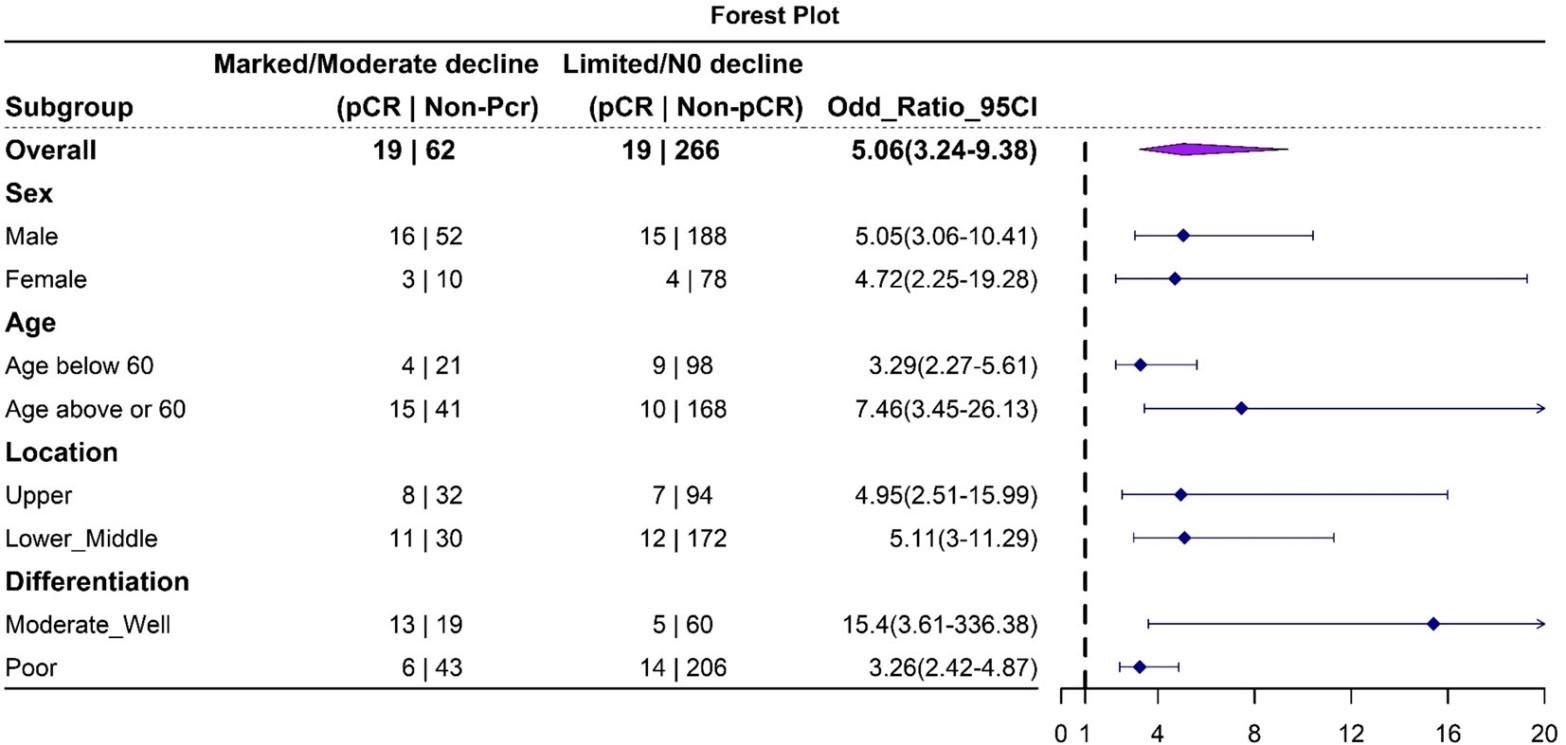
Forest plot depicting the results of subgroup analysis. The analysis indicates that this trajectory classification model is robust in predicting pathological complete response (pCR) across all subgroups.

Following neoadjuvant chemotherapy (NAC), 10.4% (38/366) of patients achieved pCR. Notably, pCR rates were strongly associated with CEA trajectory subtypes (**Figure 3**): Type I (41%), Type II (15.8%), and Type III (6.7%). Lymph node remission also correlated significantly with CEA trajectories: Type I (79.2% ypN0), Type II (35.1% ypN0), and Type III (47% ypN0) (p = 0.001). This highlights the value of CEA dynamics in predicting both pCR and lymph node remission after NAC. In the logistics multivariate analysis, with pathological complete response as the favorable outcome and all the pre-surgery clinical factors as the researching factors, the CEA trajectory subtypes and tumor differentiation are the only two independent factors that are associated with pCR, as shown in the **Figure 4**, the CEA trajectory subtypes have a significant higher weights in the model, indicating that CEA trajectory subtypes have more predictive power. As shown in the **Figure 5**, in the subgroup analysis, the predictive value of CEA trajectory subtypes remain robust across all the subgroups.

### Survival analysis

**Figure 6:**
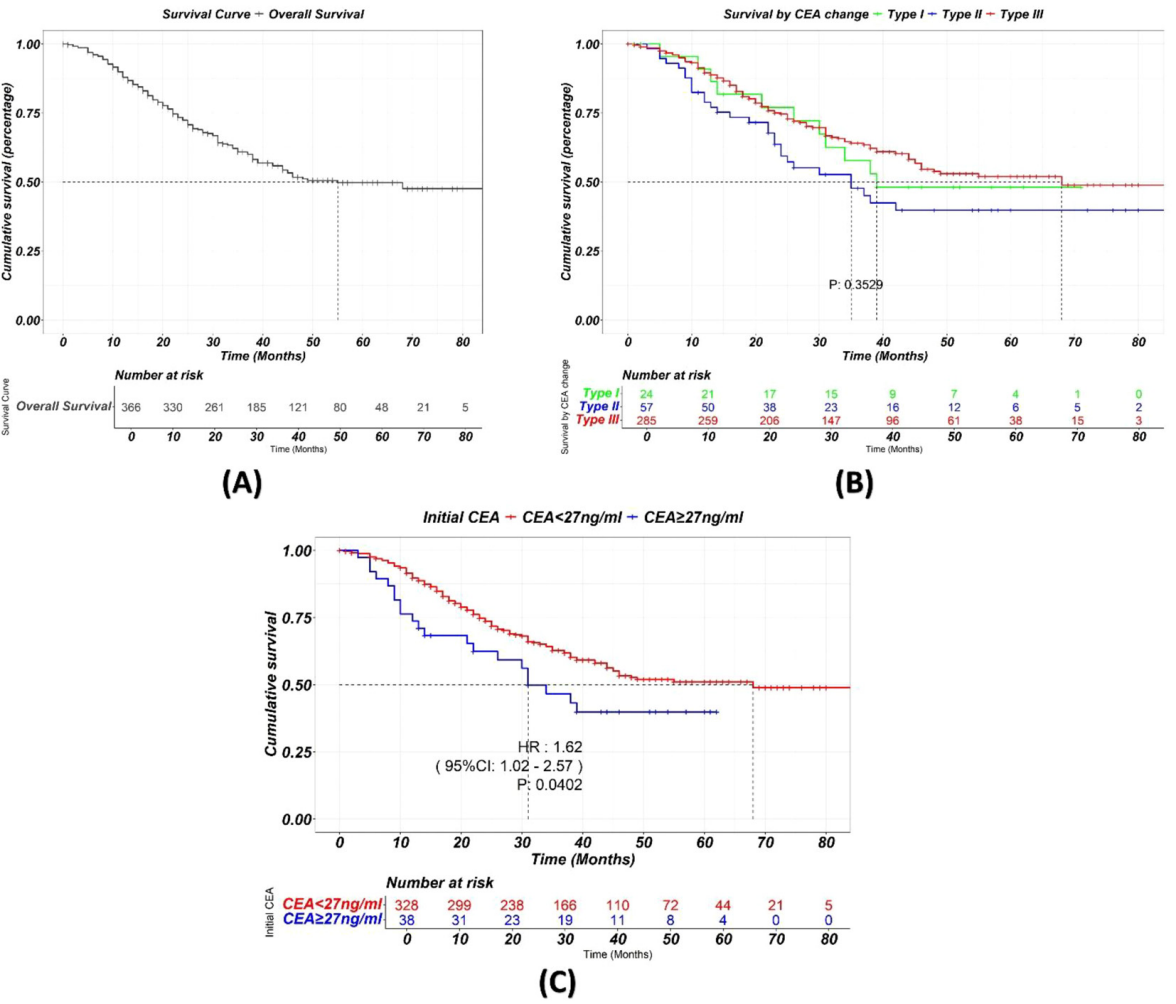
**(A)** Kaplan-Meier curve showing the overall survival of the total cohort. **(B)** Kaplan-Meier curve showing survival stratified by different trajectory classes during chemotherapy. While CEA change during NAC significantly is a strong predictor of pCR, it does not appear to significantly impact long-term survival. **(C)** Higher initial CEA levels before chemotherapy were significantly associated with worse OS.

Survival analysis (**Figure 6**) shows a median survival of 55 months for the total cohort. No significant differences in OS were found among the three CEA trajectory subtypes (p=0.3529). However, higher initial CEA levels before chemotherapy were significantly associated with worse OS (p = 0.04).

## Discussion

In this study, we found that a marked decline in CEA levels during neoadjuvant chemotherapy (NAC) is associated with a favorable pathological response. We developed a classification system, based on the magnitude of CEA decline, for predicting pathological complete response after NAC. This system is not only easy to use but also lays the groundwork for the potential development of personalized surgical strategies.

Gastric cancer remains a heavy burden on the global health system, especially in the east Asia (17). Neoadjuvant chemotherapy (NAC) has been widely utilized in the preoperative treatment settings in advanced cases, aiming to downstage tumors, improve R0 resection rate and survival. However, patients’ responses to NAC vary, with approximately 5-15% achieving pCR (4, 18). This has led to the suggestion of function-preserving surgery, such as reduced lymphadenectomy and subtotal gastrectomy, aiming to improve quality of life (19, 20). Nevertheless, several concerns remain. Firstly, research indicates that residual cancer cells may persist in perigastric lymph nodes if pCR is not achieved (11). Secondly, pCR is relatively rare. Thirdly, there are no reliable preoperative diagnostic tools to confirm pCR before surgery.

In the recent years, the emergence of immunotherapy has changed the logic of cancer management including gastric cancer (21, 22). Integrating immune checkpoint inhibitors (ICIs) into neoadjuvant treatment has achieved promising results. For example, Yuan et al. (23) reported that adding toripalimab to SOX/XELOX regimens significantly improved both pCR and major pathological response rates. Similarly, Yin et al. (24) demonstrated a 25% pCR rate with tislelizumab plus SOX, while Karukonda et al. (25) showed even greater improvement (35.7% pCR) when combining ICIs with neoadjuvant chemoradiotherapy. Preliminary Phase III trial data further substantiate this trend, with a 2-6 folds increase in pCR rates in ICI-treated cohorts compared to chemotherapy alone (26). These remarkable improvements in treatment efficacy have paved the way for the potential expansion of function-preserving surgery after NAC. However, the accurate determination of pCR before surgery remains a critical challenge.

Several studies have attempted to predict pCR after neoadjuvant chemotherapy (NAC) in AGC. Some have used pre-treatment clinical factors, such as CEA level, tumor differentiation, and lymphocyte count (27). Others have employed pre-treatment radiomic features (28) or combined deep learning with radiomics (29, 30). However, these studies focus on predicting response before NAC, that is, trying to predict chemotherapy sensitivity rather than determining the actual post-treatment tumor status. One study did evaluate post-NAC response using 68Ga-FAPI-04 and 18F-FDG PET/CT after one cycle of NAC, but this approach requires complex and costly imaging techniques, limiting its practical application (31). A simpler and more accessible method is needed.

Carcinoembryonic antigen, a 180kDa GPI-linked glycoprotein belonging to the immunoglobulin cell adhesion molecule superfamily, plays a key role in various endothelial cell functions, including adhesion, proliferation, and migration (32). CEA family comprises 29 genes, with 18 normally expressed, located on chromosome 19q13.2 (33). As a well-established tumor marker, CEA is frequently elevated in gastrointestinal malignancies (15). Several guidelines recommend its use for monitoring gastric cancer, due to its association with tumor burden and stage (34, 35). Logically, the dynamic change in CEA levels during chemotherapy should reflect the tumor’s response to treatment. To test this hypothesis, we retrieved CEA data for our study cohort, selecting values at three key time points: before, during (at an intermediate point), and after NAC. The trajectories formed by these three values represent the overall trend of CEA change during treatment. Initially, we analyzed the static CEA values at each time point and their relationship with treatment outcomes. However, no significant correlation with pathological response was observed at any individual time point. Subsequently, we focused on the change in CEA values during NAC, revealing a strong correlation. To facilitate interpretation and clinical application, we classified CEA trajectories into three subgroups based on the magnitude of change, utilizing 80% and 40% declines as cut-off points. Subgroups exhibiting marked (>=80%) or moderate (>=40% but <80%) decline in CEA values (Type I and II, respectively) demonstrated a significantly higher pCR rate compared to those with limited/no change (<40% decline or increase, Type III), highlighting the importance of the dynamic change in CEA levels during NAC rather than the static values alone.

This classification system can assist in developing personalized surgical plans for individual patients, particularly those with tumors located in the upper stomach. Traditionally, advanced-stage proximal gastric adenocarcinoma necessitates total gastrectomy (TG), which often results in poor quality of life (QOL), with 26.1% of patients experiencing severe anemia and 21.7% suffering from malnutrition (36). Proximal gastrectomy (PG) offers an alternative with improved postoperative QOL (20), but its current indication is limited to early gastric cancer with cT1 (35, 37). Although NAC can downstage tumors, concerns persist regarding inadequate remission in lymph nodes and the primary tumor. Our study demonstrates that this CEA-based classification correlates with not only a higher pCR rate but also more favorable lymph node remission. This system is robust across all subgroups, including upper stomach tumors, suggests that certain types of proximal gastric cancer patients were potential candidate for function persevering proximal gastrectomy.

Our study also found that tumor differentiation, in conjunction with CEA dynamics, was associated with a higher pCR rate. This finding aligns with previous reports, such as Chen et al., which demonstrated that well-differentiated tumors not only exhibit a better pathological response to NAC (27) but also result in improved survival outcomes (38). Combining CEA dynamics and tumor differentiation, we developed a model for predicting pCR after NAC. This model provides a practical and accessible tool for clinical decision-making.

Our study did not find significant differences in survival outcomes between different CEA trajectory subtypes. This suggests that while CEA dynamics during neoadjuvant chemotherapy (NAC) reflect tumor response and chemotherapy sensitivity, they may not be the sole determinant of overall survival. Survival in gastric cancer is influenced by multiple factors, including initial TNM stage, extent of surgical resection, postoperative complications, and adjuvant therapy (39). Chemotherapy sensitivity, as reflected by CEA dynamics, is just one contributing factor. However, we did observe a significant association between higher initial CEA levels and worse survival, highlighting the importance of initial tumor burden in determining long-term outcomes.

This study has several limitations. First, the retrospective nature of the study design inherently introduces potential biases, such as selection and information bias. Second, an external validation in diverse patient cohorts is needed to further validate the robustness of our findings.

## Conclusions

In conclusion, our study demonstrates that the trajectory of CEA change during NAC is a promising predictor of pCR in AGC. This simple and accessible model may facilitate personalized treatment decisions and contribute to the development of less invasive surgical strategies for gastric cancer patients.

## Data Availability

The raw data supporting the conclusions of this article will be made available by the authors, without undue reservation.
